# Practices of North American pediatric gastroenterologists in the management of celiac disease—A survey study

**DOI:** 10.1002/jpr3.12166

**Published:** 2025-03-10

**Authors:** Hamza Hassan Khan, Sanjay Kumar, Hernando Lyons

**Affiliations:** ^1^ Division of Pediatric Gastroenterology, Department of Pediatrics Arkansas Children's Hospital—University of Arkansas for Medical Sciences Little Rock Arkansas USA; ^2^ Department of Pediatrics Henry Ford St. John Children's Hospital Detroit Michigan USA; ^3^ Division of Pediatric Gastroenterology Henry Ford St. John Children's Hospital, Wayne State University School of Medicine Michigan USA

**Keywords:** autoimmune disorder, children, gastrointestinal disease

## Abstract

Celiac disease (CD) is a common autoimmune disorder characterized by an immune‐mediated reaction to gluten. We conducted a survey study of the pediatric gastroenterology list server to assess the practices of North American pediatric gastroenterologists in the management of CD. Overall, 160 out of 2400 respondents participated in the study, of which 52.5% of the respondents were females and 72.5% were practicing in university hospitals. Overall, respondents were practicing in adherence to the latest guidelines, except only 36% were screening for hepatitis B virus immunization at diagnosis on most of the visits, and 25% were utilizing human leukocyte antigens typing on most visits if serologies were negative. In addition, female respondents screened for vitamin D deficiency more often than males with a *p* value < 0.05.

## Abstract (highlights)

**What is Known:**

- Celiac disease (CD) is a common autoimmune disease that affects 1% of the world's population and is characterized by an autoimmune‐mediated reaction to gluten in genetically predisposed individuals.
- Most of the current studies are focused on the diagnosis with relatively little attention given to follow‐up.

**What is New:**

- To our knowledge, this is the first study to assess the practices of North American pediatric gastroenterologists in the management of CD.
- Our study found most providers adhere to the latest guidelines.

## 1 INTRODUCTION

Celiac disease (CD) is an autoimmune disorder that affects 1% of the world's population and is characterized by an immune‐mediated reaction to gluten in genetically predisposed individuals.^1^, ^2^, ^3^, ^4^, ^5^ The diverse clinical manifestations vary from asymptomatic individuals to gastrointestinal (GI) symptoms including, but not limited to, diarrhea and constipation, and non‐GI manifestations including, but not limited to, osteoporosis, arthralgia, and poor growth.^6^, ^7^, ^8^, ^9^, ^10^, ^11^, ^12^

Over the last decade, multiple guidelines have been published regarding the management of CD, but they have failed to generate consensus regarding long‐term management.^13^ In 2016, Evidence‐Informed Expert Best Practice Guidelines (EIEBPG) were published.^13^ These guidelines are based on the consensus of six expert pediatric gastroenterologists of North America, who have particular interest in CD, and review of the pediatric literature regarding CD published between 1973 and 2013.^13^ A total of 172 studies were shortlisted and reviewed to generate EIEBPG. The EIEBPG provided the grade of the evidence based on the literature search and the strength of each statement based on expert consensus. The EIEBPG were later published in 2016.^13^

With the constant advancement in medical science, it is important for physicians to be up to date with these advances and to conduct their clinical practice based on the latest guidelines, ensuring consistency of care across different providers and settings. In addition, adherence to guidelines allows for appropriate monitoring of potential complications including nutritional deficiencies and autoimmune conditions. To our knowledge, the practices of the North American pediatric gastroenterologists regarding CD have not been assessed before. Hence, the primary aim of our study was to assess the practices of North American pediatric gastroenterologists in the management of CD. A secondary aim was to assess practice variation based on demography.

## 2 METHODS

### 2.1 Study type and population

This was a cross‐sectional, survey study of all pediatric gastroenterologists who had subscribed to pediatric gastroenterology (Ped GI) list server (listserv). Since there are subscribers of the listserv who practice in other continents as well, therefore, given the aim of the study, respondents practicing in other continents apart from North America were excluded.

### 2.2 Permission from the respective institutions

Institutional Review Board (IRB) approval was obtained from Ascension Saint John Hospital IRB before starting the study. In addition, Ped GI listserv gave permission to send emails to the Ped GI listserv, free of charge.

### 2.3 Methodology

The authors noticed variation among Ped GI in the management of CD within their institution, which led to the initiative of conducting a survey study to assess the practices of North American Pediatric gastroenterologists in the management of CD. There was no formal local pilot study conducted before hand.

To assess the practices, the survey was created by the investigators consisting of a total of 22 questions. Five of the questions were based on the demographic data of the survey responder including gender, number of years in practice, continent of practice, professional involvement (clinical pathway, research pathway, combination of clinical and research pathway), and setting of the clinical practice (university hospital [UH], community hospital [CH], or private setting [PS]). The remaining 17 questions were related to clinical practices majority of which were based upon EIEBPG and consisted of six domains (bone health, hematology, endocrinology, liver, nutrition, and genetics). The response options to the 17 questions were rarely, sometimes, always. For the purpose of the survey, rarely was defined as 0%–25% of the times, sometimes was defined as 25%–75% of the times, and always was defined as 75%–100% of the times. An email was sent to each Ped GI listserv subscriber with a link to a SurveyMonkey® electronic survey. A consent letter was included at the beginning of the survey; taking the survey indicated willingness to participate and the responses to the survey were kept anonymous. Table 1 enlists the detailed questions of each domain.

**Table 1:** Summary of the practices of North American pediatric gastroenterologists who are subscribers of the pediatric gastroenterology list server.

Questions	Rarely	Sometimes	Often	Grade of evidence	Strength of statement
1. How often do you routinely screen for anemia at diagnosis?	4 (2.5%)	4 (2.5%)	152 (95%)	High	Strong
2. How often do you routinely screen for anemia at follow‐up if test results were previously abnormal?	3 (1.9%)	5 (3.1%)	152 (95%)	Low	Weak
3. How often do you routinely screen for thyroid disease at diagnosis?	13 (8.1%)	16 (10%)	131 (81.9%)	Moderate	Strong
4. How often do you routinely screen for thyroid disease at follow‐up if test results were previously abnormal?	10 (6.3%)	10 (6.3%)	140 (87.5%)	Moderate	Intermediate
5. How often do you routinely screen for vitamin D at diagnosis?	9 (5.6%)	10 (6.3%)	141 (88.1%)	Low	Weak
6. How often do you routinely screen for vitamin D at follow‐up if test results were previously abnormal?	3 (1.9%)	8 (5.0%)	149 (93.1%)	Low	Weak
7. How often do you routinely screen for other fat‐soluble vitamins at diagnosis?	87 (54.4%)	35 (21.9%)	38 (23.8%)		
8. How often do you routinely screen for other fat‐soluble vitamins at follow‐up if test results were previously abnormal?	49 (30.6%)	20 (12.5%)	91 (56.9%)		
9. How often do you routinely screen for bone mineral density at diagnosis?	102 (63.7%)	22 (13.8%)	36 (22.5%)	Moderate	Weak
10. How often do you routinely screen for bone mineral density at follow‐up if test results were previously abnormal?	93 (58.1%)	29 (18.1%)	38 (23.8%)	Moderate	Weak
11. How often do you routinely screen for ALT and AST levels at diagnosis?	14 (8.8%)	17 (10.6%)	129 (80.6%)	High	Moderate
12. How often do you routinely screen for ALT and AST levels at follow‐up if test results were previously abnormal?	6 (3.8%)	13 (8.1%)	141 (88.1%)	High	Moderate
13. How often do you routinely screen for hepatitis B virus immunization status at diagnosis?	74 (46.3%)	27 (16.9%)	59 (36.9%)	Moderate	Moderate
14. How often do you routinely screen for hepatitis B virus immunization status at follow‐up if test results were previously abnormal?	58 (36.3%)	21 (13.1%)	81 (50.6%)	Moderate	Moderate
15. How often do your patients have access to a dietician at time of diagnosis and at follow‐up?		7 (4.4%)	153 (95.6%)	High	Strong
16. How often do you recommend routine gluten‐free vitamin supplementation at time of diagnosis?	25 (15.6%)	42 (26.3%)	93 (58.1%)	Moderate	Weak
17. How often do you use HLA typing for children at risk for celiac disease but have negative serology?	59 (36.9%)	61 (38.1%)	40 (25.0%)	Moderate	Strong

*Note*: The questions are based on EIEBPG. The tables also include two columns (grade of evidence and strength of statement) based on EIEBPG. A total of 160 respondents participated in the survey.

Abbreviations: ALT, alanine transaminase; AST, aspartate transaminase; EIEBPG, Evidence‐Informed Expert Best Practice Guidelines; HLA, human leukocyte antigen.

### 2.4 Data collection and statistical analysis

We included the census of individuals who were subscribers of the Ped GI listserv at the time the survey was conducted between July 2020 and September 2020. The survey responses were collected over 2 months, and two separate reminder notifications about the survey link were sent out, each 1‐month part. Data were downloaded from SurveyMonkey®, and descriptive statistics were calculated using Statistical Package for the Social Sciences, version 27.0 (SPSS. v. 27.0). Continuous variables were described as the mean with standard deviation, and categorical variables were described as frequency distributions. The chi‐square test was used to calculate descriptive statistics for categorical variables.

### 2.5 Ethics approval and consent to participate

The study was approved by the Ascension St. John Hospital IRB (IRB Reference # 1605738). An informed consent form was included at the beginning of the survey. Taking the survey indicated a willingness to participate and subsequently publish the data. The responses to the survey were kept anonymous.

## 3 RESULTS

A total of 198 respondents completed the survey, of which 160 out of 1400 (11.4%) met the inclusion criteria. The majority of the respondents were females, 84 out of 160 (52.5%), and the mean years in practice was 12 years (range: 1–51 years; standard deviation: 11 years). The majority of the respondents were in the clinical pathway, 146 out of 160 (91.3%), followed by 9 out of 160 (5.6%) in the research pathway, and 5 out of 160 (3.1%) in the combined clinical and research pathway. The majority of the respondents were practicing in UHs, 116 out of 160 (72.5%), followed by 23 out of 160 (14.4%) in CHs, 16 out of 160 (10%) in PSs, and 5 out of 160 (3.1) in other settings. Table 1 summarizes the respondents' responses to the 17 questions pertaining to clinician practices in the management of CD. A statistically significant difference (*p* < 0.05) was noted between male and female respondents with 79 out of 84 (94.0%) female respondents screening for vitamin D deficiency at diagnosis on most visits compared to 62 out of 76 (81.6%) male respondents and 78 out of 84 (92.9%) female respondents reporting access to dietitians on most visits compared to 75 out of 76 (98.7%) male respondents. In addition, only 1 out of 23 (4.3%) CH providers reported that they are screening for bone mineral density at most follow‐ups if previous results were abnormal compared to 31 out of 116 (26.7%) UH providers, 4 out of 16 (25%) PS providers, and 2 out of 5 (40%) other providers and *p* value of 0.057. Tables 2 and 3 summarize the variation in practices based upon gender and type of practice, respectively. No statistically significant differences were identified based on professional involvement.

**Table 2:** Variation in celiac disease management practices based on gender.

Management practices	Gender	Responses			Pearson chi‐square
		Rarely	Sometimes	Always	
How often do you routinely screen for anemia at diagnosis?	Males (M)	4 (5.3%)	1 (1.3%)	71 (93.4%)	NA
	Females (F)	0 (0.0%)	3 (3.6%)	81 (96.4%)	
How often do you screen for anemia at follow‐up if previous results were abnormal?	M	1 (1.3%)	2 (2.6%)	73 (96.1%)	*p* = 0.831
	F	2 (2.4%)	3 (3.6%)	79 (94%)	
How often do you screen for thyroid diseases at diagnosis?	M	10 (13.2%)	7 (9.2%)	59 (77.6%)	*p* = 0.085
	F	3 (3.6%)	9 (10.7%)	72 (85.7%)	
How often do you screen for thyroid diseases at follow‐up if previous results were abnormal?	M	7 (9.2%)	3 (3.9%)	66 (86.8%)	*p* = 0.195
	F	3 (3.6%)	7 (8.3%)	74 (88.1%)	
How often do you screen for vitamin D deficiency at diagnosis?	M	7 (9.2%)	7 (9.2%)	62 (81.6%)	*p* = 0.049*
	F	2 (2.4%)	3 (3.6%)	79 (94%)	
How often do you screen for vitamin D deficiency at follow‐up if previous results were abnormal?	M	1 (1.3%)	3 (3.9%)	72 (94.7%)	*p* = 0.740
	F	2 (2.4%)	5 (6.0%)	77 (91.7%)	
How often do you screen for other fat‐soluble vitamin deficiency at diagnosis?	M	45 (59.2%)	16 (21.1%)	15 (19.7%)	*p* = 0.438
	F	42 (50.0%)	19 (22.6%)	23 (27.4%)	
How often do you screen for other fat‐soluble vitamins at follow‐up if previous results were abnormal?	M	24 (31.6%)	10 (13.2%)	42 (55.3%)	*p* = 0.923
	F	25 (29.8%)	10 (11.9%)	49 (58.3%)	
How often do you screen for bone mineral density at diagnosis?	M	51 (67.1%)	11 (14.5%)	14 (18.4%)	*p* = 0.501
	F	51 (60.7%)	11 (13.1%)	22 (26.2%)	
How often do you screen for bone mineral density at follow‐up if previous results were abnormal?	M	48 (63.2%)	13 (17.1%)	15 (19.7%)	*p* = 0.428
	F	45 (53.6%)	16 (19.0%)	23 (27.4%)	
How often do you screen for AST and ALT at diagnosis?	M	9 (11.8%)	5 (6.6%)	62 (81.6%)	*p* = 0.147
	F	5 (6.0%)	12 (14.3%)	67 (79.8%)	
How often do you screen for AST and ALT at follow‐up if previous results were abnormal?	M	4 (5.3%)	8 (10.5%)	64 (84.2%)	*p* = 0.339
	F	2 (2.4%)	5 (6.0%)	77 (91.7%)	
How often do you screen for hepatitis B immunization status at diagnosis?	M	30 (39.5%)	15 (19.7%)	31 (40.8%)	*p* = 0.254
	F	44 (52.4%)	12 (14.3%)	28 (33.3%)	
How often do you screen for hepatitis B immunization status at follow‐up if previous results were abnormal?	M	25 (32.9%)	11 (14.5%)	40 (52.6%)	*p* = 0.682
	F	33 (39.3%)	10 (11.9%)	41 (48.8%)	
How often do your patients have access to dietitian at the time of diagnosis and at follow‐up?	M	‐	1 (1.3%)	75 (98.7%)	*p* = 0.072
	F	‐	6 (7.1%)	78 (92.9%)	
How often do you recommend routine gluten‐free vitamin supplementation at the time of diagnosis?	M	11 (14.5%)	24 (31.6%)	41 (53.9%)	*p* = 0.346
	F	14 (16.7%)	18 (21.4%)	52 (61.9%)	
How often do you use HLA typing for children at risk for celiac disease but have negative serology?	M	33 (43.4%)	28 (36.8%)	15 (19.7%)	*p* = 0.187
	F	26 (31.0%)	33 (39.3%)	25 (29.8%)	

Abbreviations: ALT, alanine transaminase; AST, aspartate transaminase; F, females; HLA, human leukocyte antigen; M, males; NA, not applicable.

* Statistically significant *p* value.

**Table 3:** Variation in celiac disease management practices based on type of clinical practice.

Management practices	Type of practice	Responses			Pearson chi‐square
		Rarely	Sometimes	Always	
How often do you routinely screen for anemia at diagnosis?	CH	2 (8.7%)	0 (0.0%)	21 (91.3%)	NA
	UH	1 (0.9%)	4 (3.4%)	111 (95.7%)	
	PP	1 (6.3%)	0 (0.0%)	15 (93.8%)	
	O	0 (0.0%)	0 (0.0%)	5 (100%)	
How often do you routinely screen for anemia at follow‐up if test results were previously abnormal?	CH	0 (0.0%)	3 (13.0%)	20 (87.0%)	NA
	UH	3 (2.6%)	2 (1.7%)	111 (95.7%)	
	PP	0 (0.0%)	0 (0.0%)	16 (100%)	
	O	0 (0.0%)	0 (0.0%)	5 (100%)	
How often do you routinely screen for thyroid disease at diagnosis?	CH	2 (8.7%)	8 (34.8%)	13 (56.5%)	NA
	UH	8 (6.9%)	8 (6.9%)	100 (86.2%)	
	PP	1 (6.3%)	0 (0.0%)	15 (93.8%)	
	O	2 (40.0%)	0 (0.0%)	3 (60.0%)	
How often do you routinely screen for thyroid disease at follow‐up if test results were previously abnormal?	CH	3 (13.0%)	4 (17.4%)	16 (69.6%)	NA
	UH	6 (5.2%)	5 (4.3%)	105 (90.5%)	
	PP	0 (0.0%)	1 (6.3%)	15 (93.8%)	
	O	1 (20%)	0 (0.0%)	4 (80.0%)	
How often do you routinely screen for vitamin D at diagnosis?	CH	1 (4.3%)	0 (0.0%)	22 (95.7%)	NA
	UH	7 (6.0%)	8 (6.9%)	101 (87.1%)	
	PP	0 (0.0%)	0 (0.0%)	16 (100%)	
	O	1 (20.0%)	2 (40.0%)	2 (40.0%)	
How often do you screen for vitamin D deficiency at follow‐up if previous results were abnormal?	CH	0 (0.0%)	0 (0.0%)	23 (100%)	NA
	UH	3 (2.6%)	8 (6.9%)	105 (90.5%)	
	PP	0 (0.0%)	0 (0.0%)	16 (100%)	
	O	0 (0.0%)	0 (0.0%)	5 (100%)	
How often do you screen for other fat‐soluble vitamin deficiency at diagnosis?	CH	15 (65.2%)	4 (17.4%)	4 (17.4%)	NA
	UH	63 (54.3%)	25 (21.6%)	28 (24.1%)	
	PP	5 (31.3%)	5 (31.3%)	6 (37.5%)	
	O	4 (80%)	1 (20%)	0 (0.0%)	
How often do you screen for other fat‐soluble vitamins at follow‐up if previous results were abnormal?	CH	11 (47.8%)	3 (13.0%)	9 (39.1%)	NA
	UH	33 (28.4%)	14 (12.1%)	69 (59.5%)	
	PP	3 (18.8%)	3 (18.8%)	10 (62.5%)	
	O	2 (40.0%)	0 (0.0%)	3 (60.0%)	
How often do you screen for bone mineral density at diagnosis?	CH	20 (87.0%)	1 (4.3%)	2 (8.7%)	NA
	UH	70 (60.3%)	19 (16.4%)	27 (23.3)	
	PP	10 (62.5%)	2 (12.5%)	4 (25.0%)	
	O	2 (40.0%)	0 (0.0%)	3 (60.0%)	
How often do you screen for bone mineral density at follow‐up if previous results were abnormal?	CH	19 (82.6%)	3 (13.0%)	1 (4.3%)	*p* = 0.057
	UH	66 (56.9%)	19 (16.4%)	31 (26.7%)	
	PP	7 (43.8%)	5 (31.3%)	4 (25.0%)	
	O	1 (20.0%)	2 (40.0%)	2 (40.0%)	
How often do you screen for AST and ALT at diagnosis?	CH	5 (21.7%)	4 (17.4%)	14 (60.9%)	NA
	UH	8 (6.9%)	13 (11.2%)	95 (81.9%)	
	PP	1 (6.3%)	0 (0.0%)	15 (93.8%)	
	O	0 (0.0%)	0 (0.0%)	5 (100%)	
How often do you screen for AST and ALT at follow‐up if previous results were abnormal?	CH	2 (8.7%)	5 (21.7%)	16 (69.6%)	NA
	UH	3 (2.6%)	6 (5.2%)	107 (92.2%)	
	PP	1 (6.3%)	2 (12.5%)	13 (81.3%)	
	O	0 (0.0%)	0 (0.0%)	5 (100%)	
How often do you screen for hepatitis B immunization status at diagnosis?	CH	15 (65.2%)	1 (4.3%)	7 (30.4%)	NA
	UH	50 (43.1%)	22 (19.0%)	44 (37.9%)	
	PP	7 (43.8%)	4 (25.0%)	5 (31.3%)	
	O	2 (40.0%)	0 (0.0%)	3 (60.0%)	
How often do you screen for hepatitis B immunization status at follow‐up if previous results were abnormal?	CH	13 (56.5%)	0 (0.0%)	10 (43.5%)	NA
	UH	37 (31.9%)	17 (14.7%)	62 (53.4%)	
	PP	7 (43.8%)	4 (25.0%)	5 (31.3%)	
	O	1 (20.0%)	0 (0.0%)	4 (80.0%)	
How often do your patients have access to dietitian at the time of diagnosis and at follow‐up?	CH	‐	3 (13.0%)	20 (87.0%)	NA
	UH	‐	0 (0.0%)	116 (100.0%)	
	PP	‐	4 (25.0%)	12 (75.0%)	
	O	‐	0 (0.0%)	5 (100.0%)	
How often do you recommend routine gluten‐free vitamin supplementation at the time of diagnosis?	CH	4 (17.4%)	4 (17.4%)	15 (65.2%)	NA
	UH	17 (14.7%)	31 (26.7%)	68 (58.6%)	
	PP	4 (25.0%)	7 (43.8%)	5 (31.3%)	
	O	0 (0.0%)	0 (0.0%)	5 (100.0%)	
How often do you use HLA typing for children at risk for celiac disease but have negative serology?	CH	12 (52.2%)	8 (34.8%)	3 (13.0%)	NA
	UH	35 (30.2%)	47 (40.5%)	34 (29.3%)	
	PP	9 (56.3%)	6 (37.5%)	1 (6.3%)	
	O	3 (60.0%)	0 (0.0%)	2 (40.0%)	

Abbreviations: ALT, alanine transaminase; AST, aspartate transaminase; CH, community hospital; HLA, human leukocyte antigen; NA, not applicable; O, other; PP, private practice; UH, university hospital.

## 4 DISCUSSION

To our knowledge, this is the first study to assess the practices of North American pediatric gastroenterologists regarding CD. It should be noted that at the time this survey study was conducted, there were the North American Society of Pediatric Gastroenterology, Hepatology, and Nutrition (NASPGHAN) CD guidelines which were published in 2005, and the EIEBPG which were published in 2016.^13^, ^14^ Since then, the European Society for Paediatric Gastroenterology Hepatology and Nutrition (ESPGHAN) have published their guidelines in 2022.^15^

Our study's response rate was 11.4%, which was not unexpected, and comparable response rates have been reported in survey studies previously. In addition, we believe that the coronavirus disease of 2019 (COVID‐19) pandemic fatigue, at the time the survey was conducted, contributed to the low response rate.^16^ We report a balance of male and female respondents and a wide range of clinical experience of the respondents. The majority of the respondents were clinicians practicing in UH who were compliant with most of the EIEBPG clinical recommendations.

Interestingly, we do report that the majority of the respondents were screening for vitamin D at diagnosis and screening for anemia and vitamin D at follow‐up if previous results were abnormal. There was also a statistically significant difference in practices of screening for vitamin D deficiency at diagnosis among male and female respondents with more females screening for them (Table 2). There was no statistically significant difference noted in the type of the provider's practice, however more providers in CH and PS were screening for vitamin D at diagnosis and follow‐up, if previously abnormal, compared to UH respondents (Table 3). Although the strength of the EIEBPG recommendations was weak for these practices, the newer ESPGHAN guidelines recommend them.^13^, ^15^

Patients with CD have been noticed to have reduced response to hepatitis B virus (HBV) immunization.^13^, ^15^ Passanisi et al. reported a response rate of HBV immunization in the range of 47%–78%.^17^ It is not clear if this response rate is secondary to genetic factors, or other contributing factors.^15^ In addition, the HBV immunization status validity itself depends upon a number of factors including the age of the patient (e.g., if the CD is diagnosed in early childhood before the child has completed adequate immunization for HBV), whether prior history of the vaccination is documented appropriately, the possibility of developing HBV antibodies among unvaccinated individuals who are in close contact with HBV positive individuals, and the weaning of antibodies titers overtime even though the individual has received adequate HBV immunization.^17^ Antibodies more than 100 IU/mL are considered a full response, between 10 and 100 IU/mL are considered partial response and require an additional booster, and antibodies less than 10 IU/mL are considered nonresponsive and warrant repeating the three vaccinations course.^13^ An individual is considered nonresponsive if the person does not develop protective antibodies after a second complete course and this can be up to 15% of vaccinated individuals.^13^ From a public health perspective, this is a major problem as these individuals are susceptible to HBV. Hence, it is important to screen an individual with CD for HBV immunization, and give booster, or repeat the vaccination course where applicable. Both EIEBPG and ESPGHAN recommend screening for HBV immunization status in newly diagnosed CD children. However, as many as 46% of the responders were rarely screening for HBV immunization status at diagnosis (Table 1). Similarly, although not statistically significant, more female respondents were rarely screening for HBV immunization at diagnosis and follow‐up compared to male respondents, and more CH respondents were rarely screening for HBV immunization compared to UH, PS, and other respondents (Tables 2 and 3). Hence, these reported practices warrant an increase in awareness among the providers regarding adhering to the latest guidelines.

Regarding routine vitamin supplementation, although EIEBPG guidelines previously made a weak recommendation, ESPGHAN recommended nutritional supplementation in addition to gluten‐free diet since they are important for growth and neurodevelopment in children and improvement overtime may take long.^13^, ^15^ Reassuringly, more than half of our respondents were already practicing these recommendations (Table 1). Although not statistically significant, more female and CH providers were recommending gluten‐free multivitamins compared to male and UH providers (Table 2).

Moreover, EIEBPG strongly recommends human leukocyte antigens (HLAs) typing for children who have negative serology but are still at risk for CD.^13^ Similarly, ESPGHAN also recommends HLA typing in uncertain situations.^15^ This is based on the high negative predictive value of HLA‐DQ2 and DQ8.^15^ This high negative predictive value is of great clinical importance in ruling out CD in individuals with first degree relatives, as well as in individuals without a proper diagnosis who have already embrace a gluten free diet.^13^ Unfortunately, as many as 37% of our study respondents were rarely utilizing HLA typing in at‐risk population if serologies were negative (Table 1). Even though it was not statistically significant, the trends of rarely utilizing HLA typing were more among male respondents and PS respondents (Tables 2 and 3). This raises the question that either the providers are not up to date with the current guidelines or there is a concern for resources in the clinical practice they are practicing in or there has been concern regarding the level of evidence of EIEBPG.

Previously published EIEBPG made a weak recommendation regarding screening for bone mineral density if previous were abnormal; the newer ESPGHAN guidelines recommend against routine screening for bone mineral density at diagnosis; however, they recommend serial bone‐density testing every 1–2 years until normalization if a patient was previously found to have a loss of bone mineral density.^13^, ^15^ Many of the clinicians practicing in the UH were already screening for bone mineral density in patients with abnormal previous results compared to the CH (Table 3). In addition, although not statistically significant, more female providers were screening for bone mineral density at diagnosis and at follow‐up, if previously abnormal, compared to male providers (Table 2). The authors postulate that the differences in these practices between UH and CH are likely secondary to limited resources in CH compared to UH. Another possibility would be that EIEBPG made a weak recommendation and at the time this survey was conducted, ESPGHAN guidelines were not published.

Finally, access to dietitians is vital to optimize the care of patients with CD. Our study reports statistically significant differences among respondents' experiences, with male respondents reporting more access to a dietitian in their practice than female respondents. Although not statistically significant, all UH and other providers had access to a dietitian on most of their visits compared to CH and PS respondents (Tables 2 and 3). This raises concerns about discrepancies in resource distribution based on the provider's gender and type of practice.

This survey study was limited by the low response rate, response bias as busy providers may not have responded to the survey, and the distribution of the respondents as most of the respondents were from UH. Although these limitations limit the generalizability of the practices among North American pediatric gastroenterologists, authors believe that the survey was able to highlight important differences among the survey responders that require increasing awareness regarding the standard of care to ensure consistency of care across different providers and settings. The authors suggest further observational studies in the future, including surveying individuals in person at the NASPGHAN annual conference to improve the response rate, hence power, and generalizability of the data.

## 5 CONCLUSION

Overall, most study respondents were adhering to EIEBPG. It is interesting that some of the clinicians were already practicing certain practices that were not strongly recommended by EIEBPG but were later recommended by ESPGHAN. Our study found that there is room for improving screening for HBV immunization status and utilizing HLA typing for patients with negative serology or in uncertain situations. We believe this study will generate a leading point for future discussions regarding improving the practices of pediatric gastroenterologists to better understand, diagnose, and manage patients with CD. Our study was limited by the low response rate of respondents and response bias, and most of the respondents were practicing in UH, which limits the generalizability of the practices.

## CONFLICT OF INTEREST STATEMENT

The authors declare no conflicts of interest.

## Data Availability

The raw data are available upon request.
